# Human subjects protection issues in QUERI implementation research: QUERI Series

**DOI:** 10.1186/1748-5908-3-10

**Published:** 2008-02-15

**Authors:** Edmund Chaney, Laura G Rabuck, Jane Uman, Deborah C Mittman, Carol Simons, Barbara F Simon, Mona Ritchie, Marisue Cody, Lisa V Rubenstein

**Affiliations:** 1 HSR&D Northwest Center of Excellence for Outcomes Research in Older Adults, VA Puget Sound Health Care System, Metropolitan Park West, 1100 Olive Way #1400, Seattle, Washington, USA; 2 Department of Psychiatry and Behavioral Sciences, School of Medicine, University of Washington, Seattle, Washington, USA; 3 VA Center for the Study of Healthcare Provider Behavior, VA Greater Los Angeles Healthcare System, Los Angeles, California, USA; 4 HSR&D Center for Mental Healthcare and Outcomes Research, Central Arkansas Veterans Healthcare System, Little Rock, Arkansas, USA; 5 Center on Advice & Compliance Help (COACH) VA Office of Research & Development, Washington, DC, USA; 6 RAND Health Program, Santa Monica, California, USA; 7 David Geffen School of Medicine, University of California Los Angeles, Los Angeles, California, USA

## Abstract

**Background:**

Human Subjects protections approaches, specifically those relating to research review board oversight, vary throughout the world. While all are designed to protect participants involved in research, the structure and specifics of these institutional review boards (IRBs) can and do differ. This variation affects all types of research, particularly implementation research.

**Methods:**

In 2001, we began a series of inter-related studies on implementing evidence-based collaborative care for depression in Veterans Health Administration primary care. We have submitted more than 100 IRB applications, amendments, and renewals, and in doing so, we have interacted with 13 VA and University IRBs across the United States (U.S.). We present four overarching IRB-related themes encountered throughout the implementation of our projects, and within each theme, identify key challenges and suggest approaches that have proved useful. Where applicable, we showcase process aids developed to assist in resolving a particular IRB challenge.

**Results:**

There are issues unique to implementation research, as this type of research may not fit within the traditional Human Subjects paradigm used to assess clinical trials. Risks in implementation research are generally related to breaches of confidentiality, rather than health risks associated with traditional clinical trials. The implementation-specific challenges discussed are: external validity considerations, Plan-Do-Study-Act cycles, risk-benefit issues, the multiple roles of researchers and subjects, and system-level unit of analysis.

**Discussion:**

Specific aspects of implementation research interact with variations in knowledge, procedures, and regulatory interpretations across IRBs to affect the implementation and study of best methods to increase evidence-based practice. Through lack of unambiguous guidelines and local liability concerns, IRBs are often at risk of applying both variable and inappropriate or unnecessary standards to implementation research that are not consistent with the spirit of the Belmont Report (a summary of basic ethical principles identified by the National Commission for the Protection of Human Subjects of Biomedical and Behavioral Research), and which impede the conduct of evidence-based quality improvement research. While there are promising developments in the IRB community, it is incumbent upon implementation researchers to interact with IRBs in a manner that assists appropriate risk-benefit determinations and helps prevent the process from having a negative impact on efforts to reduce the lag in implementing best practices.

## Background

Human Subjects protections approaches, specifically those relating to research review board oversight, vary throughout the world. While all are designed to protect participants involved in research, the structure and specifics of these institutional review boards (IRBs) can and do differ. Central review boards, dual systems of central and local review, review boards affiliated with universities or hospitals, or un-affiliated independent review boards are used to review research protocols [1]. Within the U.S., the IRB system is struggling to effectively manage an increase in workload, specialization of scientific protocols, increased regulatory burden, and increased responsibilities beyond risk-benefit assessments to include conflict of interest, environmental impact review, HIPAA (Health Insurance Portability and Accountability Act), and others [2]. Nowhere is the result of these issues more prevalent than in IRB review of implementation research.

Implementation research in health care is characterized by a focus on evaluating evidence-based quality improvement [3] and often involves partnerships between researchers and clinicians across multiple sites [4]. As a result, implementation research faces many of the same human subjects challenges as other types of multi-site studies and research involving a quality improvement (QI) component [4].

The defining issue of human subjects protection within implementation research is the decision of whether or not implementation research should undergo IRB review. This is not always clear, particularly in studies with a QI element. There are several key determinations regarding implementation studies, beginning with a determination of whether the activity is actually research and, therefore, reviewable. Subsequent risk-benefit determinations include: whether human subjects are involved, whether the criteria for minimal risk definitions are met, what type of consent mechanisms are appropriate, and how to weigh individual risk of breach of confidentiality (often the primary risk) with potential societal benefit. These determinations derive from ethical, regulatory, and liability-related principles and demands, as well as from each particular IRB's interpretation of the risk- benefit issues given local issues and concerns. Currently, there is no official consensus on whether and how to review QI research, or other research types with QI elements, such as implementation research [5-10]. Whether appropriate or not, once a QI research protocol begins IRB review, it is thrust into a process that may not be optimal for balancing risk-benefit issues.

The number of complex, multi-site studies has increased over the past three decades, imposing a greater burden on IRBs and potentially slowing review of all types of studies. Researchers in the United States (U.S.) and elsewhere report great variability in the way IRBs evaluate multi-site protocols, time required for approval, and documentation of consent in studies [11-16]. Greene and Geiger's literature review of IRB approvals for multicenter trials and observational studies within the U.S., outlines the variability in IRB review processes and its potential effects on research [17].

McWilliams reported that 31 IRBs generated study-risk determinations from minimal to high for the same genetic epidemiology protocol, resulting in 7 expedited reviews and 24 full reviews across the sites. They reported a mean of 32 (range 9–72) days for those sites undergoing expedited review of their multi-site study, and a mean of 82 days (range 13–252) for the same protocol experiencing full review at other sites. In their study of IRB variation, Stair et al. experienced a median of 38 days for review and approval (range 26–62) [16]. This variability can result in disparities in application, be detrimental to the conduct of the study, increase the time needed to conduct the study, and increase costs [7,11,12]. The use of Centralized IRBs may remediate the issue of variability, but researchers should remain cautiously optimistic, as the literature from countries with a centralized review mechanism, such as the United Kingdom (U.K.), suggest that not all challenges faced by multi-site studies are resolved. Tod et al. report that while multi-site research in the U.K. is now overseen by Multi-centre Research Ethics Committees, this addition of central review has not eliminated the need to also seek Local Research Ethics Committee approval. Critics of the system feel that this has created "an inefficient two-tier system of ethical review" [18]. Long delays have been reported from the time of initial submission until approval. Cassell and Young raise similar concerns in their discussion of U.K. Human Subjects review [19].

Research protocols within the U.S. Department of Veterans Affairs (VA) are not immune from these challenges [20]. The VA healthcare system shares some similarities with the Canadian or British Health Care systems, in that it provides care to all veterans, but differs in that it has a tiered payment system. Some veterans qualify for total care, or total care for specific service-related conditions or disabilities, at no expense to the individual, while other veterans receive care through the VA's integrated healthcare network on a fee-for-service basis.

The purpose of this paper is two-fold. First, we address and provide approaches and process aids for common human subjects protection challenges, focusing on those relevant to implementation research, and using our research within the VA as an example. The second purpose is to support the development of future improvements in both IRB and implementation processes that will enhance research/clinical partnerships. This article is one in a *Series *of articles documenting implementation science frameworks and approaches developed by VA's Quality Enhancement Research Initiative (QUERI). QUERI is briefly outlined in Table 1 and is described in more detail in previous publications [21,22]. The *Series' *introductory article [23] highlights aspects of QUERI that are related specifically to implementation science and describe additional types of articles contained in the *QUERI Series*.

**Table 1:** The VA Quality Enhancement Research Initiative (QUERI)

The U.S. Department of Veterans Affairs' (VA) Quality Enhancement Research Initiative (QUERI) was launched in 1998. QUERI was designed to harness VA's health services research expertise and resources in an ongoing system-wide effort to improve the performance of the VA healthcare system and, thus, quality of care for veterans. QUERI researchers collaborate with VA policy and practice leaders, clinicians, and operations staff to implement appropriate evidence-based practices into routine clinical care. They work within distinct disease- or condition-specific QUERI Centers and utilize a standard six-step process:
1) Identify high-risk/high-volume diseases or problems 2) Identify best practices 3) Define existing practice patterns and outcomes across the VA and current variation from best practices 4) Identify and implement interventions to promote best practices 5) Document that best practices improve outcomes 6) Document that outcomes are associated with improved health-related quality of life.
Within Step 4, QUERI implementation efforts generally follow a sequence of four phases to enable the refinement and spread of effective and sustainable implementation programs across multiple VA medical centers and clinics. The phases include:
1) Single site pilot, 2) Small scale, multi-site implementation trial, 3) Large scale, multi-region implementation trial, and 4) System-wide roll-out.
Researchers employ additional QUERI frameworks and tools, as highlighted in this *Series*, to enhance achievement of each project's quality improvement and implementation science goals.

## Methods

In 2001, we began a series of inter-related studies on implementing evidence-based collaborative care for depression in VA primary care known by the acronyms TIDES (Translating Initiatives for Depression into Effective Solutions), WAVES (Well-being Among Veterans Enhancement Study), and COVES (Cost and Value of Evidence-based Solutions for Depression) for the initial studies, and ReTIDES (Regional TIDES) for the subsequent regional rollout (Table 2). Our VA-funded multi-site projects required a two-step approach to obtain necessary IRB approval. Because our projects represent collaboration among four regional health services research centers with Principal or Co-Principal Investigators at each of these sites, protocols had to be approved by their respective IRBs on the basis of Investigator involvement. Then each local Site Principal Investigator (PI) participating in the implementation research submitted the protocol to their IRB with our assistance. The sites we work with were either covered by their own resident VA IRB or an affiliated University IRB. We have submitted a combined total of more than 100 IRB applications, amendments, and renewals in the past six years for these four projects, and in doing so have interacted with six resident VA IRBs and seven contractually affiliated University IRBs across the United States.

**Table 2:** Description of our inter-related QUERI supported projects

**Project**	**Type**	**Participants**	**Goals**
*TIDES*	Action research partnership with formative evaluation	◆ Primary care, mental health, nursing and administrative leaders from three regional veterans health networks; ◆ Experienced depression care researchers; and ◆ Depressed veterans.	Study feasibility and safety of evidenced-based quality improvement for depression care. Steps included: 1) Adapt depression collaborative care models to VA settings through Evidence-Based Quality Improvement for Depression (EBQID); 2) Support and evaluate depression collaborative care implementation; and 3) Prepare for dissemination of depression improvement methods and materials throughout the VHA.
*WAVES*	Site-randomized controlled trial	◆ TIDES sites and matched control sites, and ◆ Veterans enrolled in primary care clinics screened for depression.	Implement & test depression collaborative care using TIDES evidence-based model.
*COVES*	Mixed method; administrative data analysis and qualitative interviews	◆ Stakeholders (patients, clinicians, administrators), and ◆ Administrators of databases of veterans' records.	Understand cost and value tradeoffs of TIDES implementation using VA cost information and semi-structured interviews with TIDES stakeholders.
*ReTIDES*	Regional demonstration project with program evaluation	All levels of VHA administrative structure including: ◆ Central Office, ◆ 4 regional health networks and administrative control networks, ◆ VA medical centers, ◆ Clinics, ◆ Providers, ◆ Administrators, and ◆ Patients.	Support progress of TIDES collaborative care model toward national VHA implementation. Sustain and spread TIDES evidence-based depression care model to additional VHA sites

In Table 3, we present the four overarching IRB-related themes encountered throughout the implementation of our projects, including items specific to implementation research, operating within multiple health care systems/sites, coordinating with multiple local site PIs, and managing differences among multiple IRBs. Within each issue, we identify key challenges and suggest approaches that have proved useful and may aid other researchers. Where applicable, we showcase process aids developed to assist in resolving a particular IRB challenge. The process aids are included as additional files and range from process guidelines through examples of explanatory material to a relational tracking database. Table 3 provides the structure and order for the paper. Many of these themes and challenges are interrelated and may overlap and/or complement each other among sections. As always, IRB review is a dynamic process that continues to change over time as new controversies present themselves, and issues that were at one time problematic become routine both within individual IRBs and in the field of human subjects protection.

**Table 3:** Implementation research themes, challenges and process aids

**Theme**	**Challenge**	**Approach**	**Process Aid(s)**
*Implementation-specific*	External validity considerations	▪ Clearly explain & support issues. ▪ Use layperson language.	▪ Implementation Evaluation Theory: Stetler Model of Research Utilization & RE-AIM
	PDSA intervention adaptation cycles	▪ Discuss with IRB and select component approach or modification approach.	
	Risk-benefit issues (minimal risk)	▪ Request exemption or expedited review. ▪ Consider opinion letters. ▪ Clearly explain & support issues. ▪ Use lay person language.	
	Multiple roles of researchers/facilitators & subject/participants	▪ Clearly explain roles and risk appropriate to each.	
	System-level unit of analysis	▪ Clearly delineate various levels, risk appropriate, and justification for each.	
*Multiple health care systems/sites*	Transparency of system/researcher ethical responsibilities	▪ Specify division of responsibilities between researchers and site clinical staff.	▪ Project Implementation Charter & Site Memorandum of Agreement
	Relationships among IRB and participating organizations	▪ Research through websites or personal contacts. ▪ Inquire about nature of oversight relationships.	▪ IRB Contact Questionnaire
	Maintaining accountability across systems/sites	▪ Centralize records retention at a single administrative site.	▪ IRB Relational Database
*Multiple local site PIs*	Recruitment	▪ Communicate responsibilities, requirements, and level of commitment.	▪ Project Implementation Charter & Site Memorandum of Agreement
	Varying levels of experience & limited time	▪ Orient to role responsibilities. ▪ Provide high level of administrative support.	▪ Site PI Orientation Checklist
	Varying ethics certification requirements	▪ Investigate required trainings. ▪ Document approved trainings.	▪ IRB Relational Database
	Varying levels of involvement	▪ Invite participation in project related activities.	
*Multiple IRBs*	Lack of standardized procedures and forms	▪ Create template text to distribute to research team.	
	Communication with IRB/R&D Administrators	▪ Consider administrators integral members of research team. ▪ Communicate multi-site nature of project.	▪ IRB Contact Questionnaire
	Multiple, competing renewal deadlines	▪ Budget time for form changes. ▪ Create deadline reports from database.	▪ IRB Relational Database
	Variable AE definitions and reporting requirements	▪ Collect definitions, reporting requirements, etc.	▪ IRB Contact Questionnaire
	Coordination & consistency in IRB related tasks	▪ Consider budgeting for an IRB Specialist position. ▪ Budget for sufficient resources. ▪ Use regular conference calls.	▪ IRB Specialist Position Description ▪ IRB Call Minute Template

## Results: The implementation research IRB experience

### 1. Implementation-specific issues

In this section, we present IRB challenges specific to implementation research. Implementation research uses a variety of methodologies at different stages and often requires collaboration among disciplines [24]. Some implementation research issues may not fit within the traditional human subjects paradigm used to assess clinical trials. Risks in implementation research are generally related to loss of confidentiality, rather than risks associated with drug treatments, as in traditional clinical trials. The implementation-specific challenges introduced below are: external validity considerations, Plan-Do-Study-Act cycles, risk-benefit issues, the multiple roles of researchers and subjects, and system-level unit of analysis.

#### a. External validity considerations

Most IRBs tend to be much more familiar with the rationales for biomedical research procedures than with those for implementation research. Two conceptual models can be helpful in providing clear explanations for specific aspects of implementation research that promote external validity, and also bear on human subjects protection issues – the Stetler Model of Research Utilization [25,26] and the RE-AIM framework [27-29]. These models can be incorporated in IRB applications by researchers, as appropriate, to inform their interactions with IRBs, as they describe external validity considerations. While these frameworks came to our attention after we had put much thought into our approach to IRB issues, they may be helpful to other researchers. These two models are presented briefly in Additional file 1: Implementation Evaluation Theory: Stetler Model of Research Utilization and RE-AIM.

#### b. Plan – Do – Study – Act (PDSA) intervention adaptation cycles

PDSA cycles are often a cornerstone of implementation research. The nature of PDSA cycles means that implementation researchers may not be able to specify all aspects of the project ahead of time for the IRB to judge risk-benefit issues in the initial application. To change a care system in a sustainable fashion, an "intervention" must be both individualized to the resources and goals of each site, and dynamic enough to take into account the inevitability of changing conditions. PDSA cycles within a particular study may incorporate multiple levels of formative evaluation [4]. When formative evaluation results are applied to the project, the equivalent of Plan Do Study Act cycles [30] result in changes that may be unforeseen at the beginning of the project.

We utilized two different approaches to address IRB concerns resulting from PDSA cycles, depending upon the preferences of each IRB. At the majority of our sites, we submitted an initial protocol that was as complete as possible, given our site knowledge and expectations, and then submitted modifications as needed. In situations where the protocol was considered exempt, we consulted with our IRB representative to ensure that the proposed modification would not alter the IRB's original decision that the protocol was exempt. However after extensive discussion and collaboration, other IRBs requested dividing the initial protocol into key component submissions, each individually well specified.

Under this approach, we submitted the first study component along with an overview of the entire project. We then submitted additional project activities for individual review in the context of the project overview. This approach allowed IRB reviewers to judge project activities in context without having to judge all components at once. Reviewers had complete information for each component at the time of submission. It allowed the level of IRB review (i.e., exempt versus full) to be appropriate to a specific component. At one study site, four consecutive components were submitted individually and received different IRB approvals.

Component 1 involved carrying out an expert panel to decide how to best administer a depression collaborative care project and was exempted. Component 2, staff qualitative interviews, was also exempted. Component 3, a survey of site clinician opinions, was not exempted but received a standard review and Waiver of Documentation of Consent. The final component, a clinical decision support software tool developed by study participants (not researchers), was acknowledged by the IRB as having been received, but not reviewable, i.e., not research.

Over time, this component solution proved to be administratively burdensome to both the IRB and the research team due to the need to track each component as a separate application, complicating modifications and renewals. We anticipate that future IRB developments will identify the best methods for introducing the unique features of individual components to the IRB and, thereby, will decrease the burden on both researchers and the IRB [9].

#### c. Risk-benefit issues

Implementation research often involves both evidence-based interventions carried out by clinical partners and evaluation activities carried out by researchers. In QUERI implementation research, the best practices to be implemented are not experimental as earlier QUERI research phases have already documented the need for clinical improvement and provided an evidence base (Table 1). Unless carefully explained and supported, we have found that it may not be obvious to IRBs that the "research" issue is not the efficacy of the best practice, but the ability of a quality improvement strategy to introduce or improve a system's ability to apply the evidence-based practice. IRBs may not realize the extent to which quality improvement principles and requirements guide clinical activities carried out in implementation projects and, therefore, risk-benefit issues might be most appropriately considered in a QI framework. Currently, the determination as to whether an activity is reviewable research or non-reviewable QI is made by the IRB. Unfortunately there is not yet a standard method for assisting an IRB to make this decision.

In preparing our applications, to assist the IRB, we sought to use clear layperson terminology to explain our project. For example:

*There are two aspects to this project that could be described as "intervention:" 1) the system-level evidence-based quality improvement process that has the goal of changing healthcare provider behavior, and 2) the depression care that the providers deliver. This project studies the former. The latter, described as collaborative care for depression, has been extensively studied and is accepted as best practice care. No experimental treatments are employed in this project*.

We have had some success in using an opinion letter process as an initial step in the application process. For example, one IRB (Table 4, Site I) considered a protocol to fall under quality improvement and be classified as "non-human" research, thus not requiring IRB review. However, for the same protocol another site required a full submission. Given the societal health benefits likely to be associated with researcher involvement with clinical systems and practices, and the known risks of poor quality care, the ethical issues of adding burden to the process of improving care should be considered in these determinations [31,32].

**Table 4:** TIDES/RETIDES IRB initial application information

**TIDES Study**				
Site*	*IRB type*	*IRB required changes?*	*Approval Type*†	*Time Elapsed‡*

A	VA in-house	No	Exempt	32
B	VA in-house	No	Exempt	67
C	University	Yes^1^	Expedited	75
D	University	No	Expedited	9
E	VA in-house	Yes^2^	Expedited	5
F	University	Yes^3^	Full	7
G§	VA in-house	No	Full	82
H¶	University	Yes^4^	Expedited	38
I	University	No	Expedited	5
				Average = 35.55

**ReTIDES Study**				

F	University	Yes^5^	Full	22
I	University	N/A	Non-human research	5
J	University	No	Exempt	3
K	University	No	Exempt	20
H¶	University	Yes^6^	Expedited	30
L	VA in-house	Yes^7^	Expedited	14
A	VA in-house	No	Exempt	6
M	VA in-house	No	Exempt	14
				Average = 14.25

* Site identification codes are uniform, showing both new sites for ReTIDES and some TIDES sites not (yet) participating in ReTIDES.

† We experienced no variance between review type preferred and actual approval type granted.

‡ In days, starting with submission date, ending with approval date (weekends, holidays included).

§ From IRB G Investigator Guide & Instructions booklet: "No research is exempt from review and there are no "expedited" reviews."

¶ This was the only site that required an electronic submission.

1 Concerns included: consent form alterations, include contact information in mailing, survey data storage, patient information protocol, number of interviews, and adding written consent at local level.

2 Concerns included: changes to allow better informed consent decision-making, protocol on frequency of subject contact, advance phone call consent, telephone interview protocol, recording interview protocol, time estimated for interviews, why patients were being interviewed, and obtaining consent.

3 Concerns included: consent obtained to access patient data registry, justification for exemption of worksheet data forms, how does VA encrypt medical records, number discrepancy, terminology issues, protocol regarding the movement of paper data, oral consent procedure, clarification of anonymous/confidential, changes to worksheets, who has data access, confirm study codes are not SSN, how is questionnaire used, and typographic errors.

4 Concerns included: typographic errors and further justification for no consent.

5 Concerns included: coercion as possible risk, determining coordinating center, providing a consent form if requested by subject, and how participation is/is not related to job description, including text about participating not affecting job security.

6 Concerns were administrative and related to the electronic submission process.

7 Concerns were administrative.

Risks in implementation research are often elusive, and the regulatory definition of "minimal risk" is imprecise. There often are no physiological risks in implementation research and, in the case of our studies, minimal psychosocial risk, including psychological stress from completing staff interviews or surveys and evaluation discomfort. Thus, implementation research might often be categorized as minimal risk. However, breach of confidentiality due to a security breach or human error remains a risk even in minimal risk studies. Researchers should accurately assess the associated risks of the research, including the evidence-based practice.

Some systems provide expedited review for research judged to be minimal risk. For example, our protocols include evaluation through surveys, medical record reviews, and stakeholder interviews. However, these multiple activities may result in complex IRB decision-making and risk to the implementation activity timeline, if after initial review the IRB decides that the study is inappropriate for expedited review, and the study must be transferred to the full-review mechanism. For example, one site (Table 4, IRB C) stated, "If the survey was the only thing that came to the IRB board, it could have been exempt or if the chart data had come to the IRB separately, it too, could have been expedited. But because it came all together, it would be hard to expedite it as a whole with all the different components presented."

Finally, if an IRB judged the initial proposal to be exempt, it became difficult to review a later modification of the proposal. For example, this occurred when a staff survey planned as an anonymous QI activity could not be feasibly carried out without participant identifiability. It then required extra effort to return to IRBs that had judged the survey as exempt, in order to notify them of the new circumstances.

#### d. Multiple roles of researchers/facilitators & multiple subject/participant classes

Sometimes it is not the implementation research practice itself that is the risk, but ill-defined relationships among researchers and the system with which they are working that may add risk. Implementation researchers may best be understood as facilitators or consultants within their research projects, with no direct decision-making authority in the intervention. Within our projects, researchers were available as consultants to local site champions who provided leadership for customizing and adapting the intervention to the sites. Researchers also functioned as consultants at the national level to encourage national rollout of improved depression care. These different roles (and risks) need to be clearly communicated with administrators and IRBs. For example, because our projects involved depression treatment, it was important to establish suicidality risk-response protocols specifying activities of researchers and clinicians of which all parties were aware.

Subjects, more appropriately called "participants," in implementation research have different relationships with researchers depending on their relationship to the health care system in which the implementation takes place. Participants may include providers, regional and/or medical center leadership, and/or patients. Patients, for example, may not encounter researchers, but have specific aspects of their medical records examined for evidence that is relevant to the implementation. Providers are crucial in the success of clinical improvement efforts, however their role as a "subject" must be carefully considered. If they see themselves as subjects from whom consent is required for their participation, their buy-in and interest in sustaining the improvement after the investigative phase is over is likely to be vitiated. All classes of participants, or their representatives, may be involved as decision-makers in the specifics of the implementation. For example, our project start-up meetings often included nurse depression care managers, primary care physicians, mental health practitioners, leadership/management personnel, and patient representatives. Clearly defining these different roles and the risk appropriate to each will enable the IRB to make an informed decision.

#### e. System-level unit of analysis

In these projects, like similar implementation efforts, patients, providers and/or sites can be units of analysis to measure/support the external validity of the implementation effort, as well as to monitor its clinical and organizational outcomes. The impact of implementation research is often assessed on the patient level through patient administrative records; at the provider level through survey results and aggregated, de-identified data; and at the site level though cost analyses, site-level performance measurements, interview results, and project records (i.e., conference call minutes, emails, etc.) analyses. IRBs that are familiar with evaluating clinical trials may not understand the necessity of collecting information at each of these levels. Each of these levels has its own set of concerns and ethical issues. For example, whereas breach of confidentiality may be a major risk for patients, concern that results of a survey may be accessible to supervisors and used for evaluative purposes may be an issue for staff. It is important to clearly delineate these various roles, the risk appropriate to each, and the justification for each when communicating with the IRB. Using clear layperson language and/or tables to present relationships may be useful.

### 2. Multiple health care systems/sites

This section focuses on concerns surrounding working with multiple sites or health care systems, namely the ethical responsibilities of researchers, preserving accountability across sites, and relationships among IRBs and other organizational entities.

#### a. Transparency of system/researcher ethical responsibilities

As consultants, researchers often function at the behest of the organizational entity. Organizational participants may hold considerably more power than the researchers by virtue of their decision-making authority. Clinicians may encounter implementation researchers as consultants, purveyors of Continuing Medical Education and/or as gatherers of information regarding professional opinions and knowledge that may be relevant to the implementation project. IRBs are appropriately concerned when researcher/clinician roles are complex, in that this can raise issues of possible coercion in recruitment of patients and staff, and/or confidentiality problems in keeping clinical, job performance, and research data separate.

We developed an Implementation Charter (see Additional file 2: Project Implementation Charter and Site Memorandum of Agreement) with participating VA regional networks that specified the division of responsibilities between researchers and site clinical staff. For example, the Charter indicated that we were responsible for training depression care managers within the clinics, but that the care managers were hired and supervised by appropriate clinical personnel. It also specified our adherence to clinical privacy rules for aspects of our project that were quality improvement, and the need for site participants who wished to utilize any QI data from the project for research purposes to seek IRB approval. This process aid was successful in clarifying our roles and expectations with participating sites.

#### b. Relationships among IRBs and participating organizations

Implementation researchers often need to satisfy multiple sets of organizational reviews and requirements before beginning a project. For example, within the VA system, a research study conducted by VA personnel and/or involving VA patients cannot begin without both IRB approval and approval by the appropriate local VA Research & Development (R&D) Committee. While the IRB reviews the protocol to ensure adequate protection of human subjects, the local R&D committee provides the primary executive review for their site, assessing the overall scientific merit of the protocol. R&D application procedures and protocols are not uniform across sites.

We researched a new study site's local IRB and R&D through web sites or personal contacts. To confirm that we had the correct IRB forms for a particular submission, we informally "interviewed" the IRB administrative contact using our IRB Contact Questionnaire (see Additional file 3: IRB Contact Questionnaire). Through this process we obtained necessary information about the particular IRB process, including their relationship with the local R&D Committee. This process enabled us to effectively submit our applications with minimal problems and demonstrated our willingness to cooperate with local administrators and procedures.

#### c. Maintaining accountability across systems/sites

As a site was brought onto a particular project, responsibility for that site was assigned to specific research personnel within the project. This created continuity within the submission process and in interactions between the IRB and the project as a whole. Record retention has been centralized for the projects. Study personnel forward electronic and/or hard copies of all IRB paperwork and communications to the administrative site in addition to keeping copies at their respective sites. As the number of sites increased across projects, it became clear that a relational database was necessary to efficiently track all the paperwork and deadlines.

Using Microsoft ACCESS, information gathered from the IRB Contact Questionnaire is entered into the database (see Additional file 4: IRB Relational Database). As applications are submitted and approvals received, additional key information is entered. The structure allows monitoring expiration deadlines, duration between submission and IRB approval, and time elapsed since submission. Information for all sites or specific to project, site, or region can be extracted and presented as reports. The database is especially useful to track information at sites where the component submission approach is utilized.

### 3. Multiple local site PIs

Enthusiastic, informed and active Site PIs are crucial to the success of a multi-site study. The study staffs' ability to finesse individual Site PI situations, accommodate the desired level of Site PI involvement, and, generally, limited local support staff availability is crucial for the project to be successful. As would be expected, we experienced several challenges collaborating with multiple Site PIs, such as recruitment, varying levels of experience and limited time, inconsistent ethics certification requirements, and varying levels of commitment to the study.

#### a. Recruitment

We have used a variety of strategies when identifying and recruiting local Site PIs. Through our contacts as researchers and established Site PI contacts, we were able to obtain information on a potential Site PIs' availability and overall interest. The initial conversation between the project PI and potential Site PI covered background information on the project, noting other clinicians who may be participating in the project, and a brief overview of responsibilities. An assigned member of the research staff followed up with the Site PI to further address questions. Communicating Site PI responsibilities, requirements, and level of commitment clearly and accurately is necessary for success. We used the Implementation Charter mentioned earlier to help clarify the role of the Site PI in relation to the researchers (See Additional file 2: Project Implementation Charter and Site Memorandum of Agreement). It is critical that the involvement of the site PI is supported by their organizational supervisors and relevant to their own career goals.

#### b. Varying levels of experience & limited time

After recruitment, the research team must assess the experience level of Site PIs with regard to their local IRB, the IRB process and responsibilities, and our project(s). One consistent factor among our Site PIs was their busy schedules and limited availability. As a result, we developed an efficient, concise protocol to gather this information through our Site PI Orientation Checklist (see Additional file 5: Site PI Orientation Checklist). Upon completion of the Checklist, roles and responsibilities of the research staff and Site PI were clearly delineated, including who would handle the majority of IRB paperwork. At some sites, research staff effectively became the research coordinator for the local Site PI. A Site PI's willingness to assist in problem-solving proved to be critical to the timeliness with which each site progressed through the IRB process. For example, some IRBs do not include remotely-based research staff on correspondence relating to the project. Site PIs are encouraged to proactively forward those documents to their research staff contact person to ensure optimal project responsiveness to IRB requirements and to minimize project delays.

#### d. Varying ethics certification requirements

All researchers are responsible for fulfilling any necessary ethics certification and training requirements prior to the start of any research. However, different sites may require renewals of these trainings at different times and may require additional certification. It is in the project's interest to monitor these requirements to avoid any project delay due to a lapse in training certification. The relational database mentioned above can be used for this purpose (see Additional file 4: IRB Relational Database).

#### d. Varying levels of involvement

Site PIs demonstrate varying levels of involvement to their role within the project. Some take an active role in communicating new ideas and suggestions regarding implementation, or independently promoting and presenting the project at conferences or meetings with clinicians. To help provide incentive, we try to involve interested Site PIs in project-related activities, such as workgroup meetings. Several of these working groups have developed and published manuscripts.

### 4. Multiple IRBs

For researchers who have the need, the logistics of working with multiple IRBs are discussed below. These include: lack of standardized procedures and forms, communication with IRB administrators, multiple competing renewal deadlines, variable adverse event reporting requirements and definitions, and issues with coordination and consistency of IRB-related tasks.

#### a. Lack of standardized procedures & forms

Our experience is that IRB procedures, forms, deadlines and meeting schedules vary from one IRB to the next. Areas of variability include: whether the IRB will communicate directly with staff other than the Site PI, structure and organization of application forms, deadlines and meeting schedules, and reporting requirements for adverse events. This diversity increases the burden on research staff. We developed a consistent approach to submissions. One senior member of our research staff composed the "template" text that was subsequently distributed to other research staff members, who tailored the text to best fit a particular IRB.

#### b. Communications with IRB and R&D administrators

The multi-site nature of our projects means that in the majority of cases, the project PI, Site PI, and research staff are in different geographical locations. Communicating this to respective administrators enables them to understand the constraints of the situation and, in some cases, resulted in their exceptional assistance in the submission process. For instance, one R&D Administrator offered to collect the necessary Site PI signatures for submissions, hand deliver the paperwork to the IRB, and inform the research team via e-mail.

At the start of the projects in 2001, many IRBs did not have websites, or if they did, all necessary forms and instructions were not available on the site. This required the assistance of the site's IRB staff in order to obtain all forms and any additional relevant information on the submission process. Today, even with many forms and instructions available online, it is crucial to communicate with IRB staff to ensure the correct forms are used to fit a particular activity (see Additional file 3: IRB Contact Checklist). Considering IRB and R&D staff as integral members of a QI effort helps minimize communication difficulties and facilitate the sometimes cumbersome IRB process.

#### c. Multiple competing renewal deadlines

Carrying out projects involving multiple IRBs creates a revolving door of renewal deadlines. This requires that the research team monitor the approval status of each site. Renewal forms change over time as IRBs revise old questions and add new ones. It is important to take this into consideration when budgeting the amount of time needed for IRB activities.

All of our IRB submissions are tracked through the relational database, and bi-weekly submission deadline reports are created that are shared with other coordinating administrative sites (see Additional file 4: IRB Relational Database) using group emails and IRB-specific conference calls.

#### d. Variable adverse event definitions & reporting requirements

Implementation projects involve the possibility of adverse events that, although not directly connected with study activities, may need to be reported to involved IRBs. Since our projects work with a depressed population, we prepared for the eventuality of a serious or successful suicide attempt. We discussed this potential risk in IRB applications and collected different IRB definitions, reporting requirements, and related items for Adverse Events (AEs) and Serious Adverse Events (SAEs) (see Additional file 3: IRB Contact Questionnaire).

There is a significant degree of variation in AE/SAE definitions and reporting requirements illustrated by the following hypothetical situation of a completed suicide by a study patient. One IRB would consider this a reportable SAE, because a subject died. A second IRB would consider it a reportable SAE because suicide is an expected risk among this study population. A third IRB would not consider it a reportable event because it was believed not to be related to the study procedures, since all established medical center and research policies and procedures were appropriately followed.

#### e. Coordination & consistency in IRB-related tasks

All the process aids and approaches mentioned above can not be fully realized without coordination, consistency, advanced planning and consultation. For example, while developing the IRB portion of our grant applications, we consulted with several IRB experts. IRB work for implementation projects is a significant resource allocation and budget issue. It may be cost-effective to budget for an IRB specialist to assist research staff. This specialist should have extensive knowledge of Human Subjects protection issues and the IRB system in which the study takes place (see Additional file 6: IRB Specialist Position Description). In our case, other project staff devoted approximately 1.5 FTEE (Full time employment equivalent) to IRB-related tasks over all study years (6 years, at present), with significant involvement both by investigators and research coordinators.

Regular conference calls were established to anticipate and discuss IRB issues within the projects. These calls ensure that research staff can discuss and resolve IRB concerns (see Additional file 7: IRB Call Minutes). While this effort is substantial, similar projects should anticipate relevant IRB challenges and budget appropriately.

## Discussion

The IRB system in the United States is overburdened, affecting all types of research, particularly implementation research. Specific aspects of implementation research interact with variations in knowledge, procedures and regulatory interpretations across IRBs to impact the implementation and study of best methods to increase evidence-based practice. Through lack of unambiguous guidelines and local liability concerns, IRBs are at risk of applying both variable and inappropriate or unnecessary standards that may impede the conduct of evidence-based QI implementation research because they are not consistent with the spirit of the Belmont Report, a summary of the three basic ethical principles (Respect for persons, Beneficence, and Justice) identified by the National Commission for the Protection of Human Subjects of Biomedical and Behavioral Research. However, there are promising developments in the IRB community. For instance, in November 2005, the Office for Human Research Protections (OHRP) sponsored a workshop on alternative models for IRB review, including Centralized IRBs, with a follow-up conference in 2006 [33,34]. Efforts to accredit IRBs may increase uniformity and the IRB's ability to be a full partner in improving care. In the meantime, it is incumbent upon implementation researchers to interact with IRBs in a manner that assists appropriate risk/benefit determinations and helps prevent the process from having a negative impact on efforts to reduce the lag in implementing best practices.

## Conclusion

IRBs are essential participants in moving implementation research forward. Our experience shows that ethical dilemmas posed by implementation research can be solved if the level of commitment and communication among stakeholders is sufficient. Our experience also highlights the high costs of working with IRBs in multi-site projects, and the necessity of developing organized, efficient ways of dealing with these issues if researchers are to participate in improving clinical quality.

## Authors' contributions

This paper is based on the IRB experiences of a series of inter-related depression studies of which EC and LR were the lead Investigators. The conceptual design of this paper was created by EC and LR with input from fellow authors and the paper was jointly written. Process aids were designed by EC, LGR, JU, DM, CS, BS and MR. The literature review for this article was carried out by EC, LGR, JU, DM, CS, and BS. MC advised with regard to IRB issues for the studies on which this paper is based and consulted on IRB issues in the VA generally. All authors read and approved the final manuscript.

## Supplementary Material

Additional file 1 Implementation evaluation theory guidance. This process aid discusses the Stetler Model & RE_AIM model and how they might be useful within the framework of Human Subjects Issues for implementation research. Click here for file

Additional file 2 Project implementation charter and site memorandum of agreement. A set of process aids that can be used to clarify the division of research roles. Click here for file

Additional file 3 IRB contact questionnaire. A process aid designed to simplify the communication and interaction with the IRB by centralizing some basic information on the IRB and its process and procedures. Click here for file

Additional file 4 IRB Relational Database. A process aid which is an example of how a relational database can be used to assist with IRB tracking across sites. Click here for file

Additional file 5 Site PI orientation checklist. A process aid to collect contact information and preference for the site PI as well as a tool for discussing IRB responsibilities and division of labor. Click here for file

Additional file 6 IRB specialist position description. a process aid job description including how having a designated person in this role can be helpful. Click here for file

Additional file 7 IRB Call Minutes Template. A template/process aid which can be used to record the discussion and particularly the decisions made during IRB calls. Click here for file
