# Supplementary material for: Human subjects protection issues in QUERI implementation research: QUERI Series
Source: Implement Sci. 2008 Feb 15;3:10. doi: 10.1186/1748-5908-3-10 (PMC2276514; doi:10.1186/1748-5908-3-10)
Supplement: Additional file 1 — Implementation evaluation theory guidance. This process aid discusses the Stetler Model & RE_AIM model and how they might be useful within the framework of Human Subjects Issues for implementation research. [file 1748-5908-3-10-S1.doc]

**Implementation evaluation theory guidance:**

**Stetler Model of Research Utilization & RE-AIM**

Purpose: These conceptual models can be helpful for internal review board (IRB) submissions by providing explanations for specific aspects of implementation research and clarifying the potential risks and benefits associated with this type of research.

Relevance: IRBs are most familiar with biomedical research procedures and may need to be oriented to the implementation research framework. These models may facilitate helpful dialogue on an implementation research protocol with an IRB.

*These process aids have been developed and refined over the course of our projects, and we anticipate they will continue to evolve over time.  Please feel free to use or adapt them to your projects as necessary.*

**Stetler Model of Research Utilization [1, 2]**

The Stetler model of Research Utilization consists of five phases that guide:

**Phase 1.** the selection of research evidence;

**Phase 2.** formal utilization critique of studies;

**Phase 3.** consideration of research findings in the context of other forms of evidence, fit to the setting that is considering implementation, alignment with current practice and feasibility of adoption;

**Phase 4.** the type of use decision and specifics of implementation; and

**Phase 5.** dynamic evaluation, the details of which depend on the use decision.

The model formally mentions the role of IRBs in Phase 5, but we suggest the researcher consider the IRB as an “influential, external factor” to be considered along with other factors beginning in Phase 1.

Considering the IRB in this light motivates many of the process aids we discuss that are designed to: 1) respond to the IRB’s information needs and processes, as well as informing decision-makers and facilitators; and 2) guide decision-making as to the content of the application to encourage facilitative IRB processes in implementation of evidence-based quality improvement.

**RE-AIM Framework [3-5]**

The RE-AIM framework (Reach, Effectiveness, Adoption, Implementation, and Maintenance) identifies information sources that are needed to promote external validity and, therefore, the types of interaction among researchers and participants that will be required to obtain that information.

The REACH of an implementation study is determined by comparing participants of all categories (i.e., administrators, clinicians and patients) with eligible non-participants within participating sites. Measurement of reach requires information about non-participants (an IRB issue) and is affected by inclusion and exclusion criteria, and by research consent. Reach is adversely affected by factors that reduce the participant pool in relation to individuals for whom the intervention to be implemented may be a best practice.

EFFECTIVENESS applies to both individuals and organizations and requires documentation and measurement of outcomes, both positive and negative. For implementation studies, this can involve the following issues:

- Documenting variability in individual outcomes often requires the ability to merge data for individual participants across several databases, usually requiring at least the transitory ability to assemble crosswalks that may be indexed by identifying information, such as social security numbers.
- Documenting any negative outcomes to individual participants is consonant with the need to report adverse consequences to IRBs.
- Documenting organizational outcome variability requires sites as units of analysis, and the study design should allow for measurement of potential negative effects at the organizational level.

ADOPTION also applies to both individuals, as “intervention agents,” and organizations. Research enrollment and consent processes that differ from routine organizational procedures may reduce the ability to examine adoption by individual participants. Similarly, using volunteer sites may restrict participation in the project to settings with a culture of innovation. Adoption at the system level requires ownership and integration into usual care processes.

The RE-AIM framework makes explicit the assessment of fidelity to the model being implemented and documenting the costs of IMPLEMENTATION. Both of these measurement areas require collecting information on participants’ activities. In addition, the Stetler model suggests that concurrent assessment of facilitators and enhancers of change at the local level are necessary to optimize achievement of the targeted goals.

Finally, documenting MAINTENANCE involves both individual and site-level data, the former of retention and maintenance or decay of individual participant outcomes attributable to the implementation and the latter, continued employment of the implemented practices by the sites and long term fidelity to the evidence base.

**References**

1. Stetler CB: **Refinement of the Stetler/Marram model for application of research findings to practice.** *Nurs Outlook* 1994, **42:**15-25.

2. Stetler CB: **Updating the Stetler Model of research utilization to facilitate evidence-based practice.** *Nurs Outlook* 2001, **49:**272-279.

3. Green LW, Glasgow RE: **Evaluating the relevance, generalization, and applicability of research: issues in external validation and translation methodology.** *Eval Health Prof* 2006, **29:**126-153.

4. Glasgow RE, McKay HG, Piette JD, Reynolds KD: **The RE-AIM framework for evaluating interventions: what can it tell us about approaches to chronic illness management?** *Patient Educ Couns* 2001, **44:**119-127.

5. Bull SS, Gillette C, Glasgow RE, Estabrooks P: **Work site health promotion research: to what extent can we generalize the results and what is needed to translate research to practice?** *Health Educ Behav* 2003, **30:**537-549.
