# Supplementary material for: Human subjects protection issues in QUERI implementation research: QUERI Series
Source: Implement Sci. 2008 Feb 15;3:10. doi: 10.1186/1748-5908-3-10 (PMC2276514; doi:10.1186/1748-5908-3-10)
Supplement: Additional file 2 — Project implementation charter and site memorandum of agreement. A set of process aids that can be used to clarify the division of research roles. [file 1748-5908-3-10-S2.doc]

**Project implementation charter and**

**site memorandum of agreement**

Purpose: These two items can be used together or separately to specify the division of responsibilities between the researchers and site clinical staff. This clarifies the roles of researchers and site clinical staff, including Site Principal Investigators (PIs).

Relevance: Clarification of roles in implementation research is necessary to understand where ethical responsibilities lie and allows an IRB to appropriately assess human subjects issues relating to possible coercion in recruitment and/or confidentiality issues. This is particularly important with regard to the role of site staff, which may be that of both collaborator and informant/subject.

*These process aids have been developed and refined over the course of our projects, and we anticipate they will continue to evolve over time.  Please feel free to use or adapt them to your projects as necessary.*

**Project YY implementation charter**

| **Decision** | **Specifics** |
| --- | --- |
| **Site XX leadership decisions** | |
| Review project YY implementation charter. | Adopt or adapt |
| Designate clinic(s) to receive depression care manager (DCM) services and expand to additional clinics as possible. | To be announced |
| Designate site PI and specify responsibilities. | To be named:   - Oversee human subjects protection issues, - Provide liaison to IRB, - Coordinate with research team on IRB issues, and - Monitor program productivity and outcomes. |
| Designate local champion(s) and specify responsibilities. | To be named:   - Provide education on value of project YY and how program works, - Collaborate with colleagues to customize project YY to best fit local clinic conditions, and - Monitor program productivity and outcomes. |
| Designate mental health leader and specify responsibilities. | To be named:   - Ensure mental health specialty (MHS) collaboration with PC, - Ensure clinical supervision of DCMs by MHS, - Submit DCM performance reviews to nursing leader, - Ensure clinical updating of MHSs and PCPs regarding depression care, - Ensure initial education and orientation of new MHS staff to project YY process, and - Monitor program productivity and outcomes. |
| Designate primary care leader and specify responsibilities. | To be named:   - Ensure PC collaboration with MHS, - Submit DCM performance reviews to nursing leader, - Ensure education of MHSs and PCPs about the project YY care model, and - Monitor program productivity and outcomes. |
| Designate nursing leader and specify responsibilities. | To be named:   - Ensure DCM integration into nursing service, in addition to PC and MHS, - Administratively supervise DCMs, - Solicit performance feedback from PC and MHS, and - Submit DCM performance reviews to nurse executive. |
| Specify depression care team members and administrative structure, including implementation sequence. | - Include PCPs, DCMs, medication managers, counselors, clinical supervisors, etc. |
| **Site XX administrative decisions** | |
| Identify target population and exclusion criteria. | - Target population may include patients who screen positive, those with a depression diagnosis, etc. - Exclusion criteria may include bipolar disorder, PTSD, etc. |
| Specify nature of collaboration between PC and MHS. | *Availability of MHS to depressed patients cared for by the DCM/PCP for:*   - urgent problems, - informal “curbside” consults on MH issues, - weekly session for DCM case load review (MHS recommendations and workload captured via CPRS group notes), - education and updating sessions on depression care, - cognitive behavioral therapy groups, and - ongoing care of complex depressed patients.   *Availability of PCPs to depressed patients cared for by DCM/MHS for:*   - urgent medical problems, - informal “curbside” consults on medical issues, - depression detection and DCM consult, - review of DCM assessment, and - initial triage/treatment decision and orders   ongoing care of medical conditions. |
| Specify treatment guidelines. | - Protocol for treatment resistant patients, - Guideline for mental health referrals, - Guideline for disengaging patients from Project YY, and - Medication management algorithm. |
| Specify suicide protocol. | - Protocol for assessing suicide risk and ensuring safety of high risk patients. |
| Identify or develop implementation tools. | - Clinical assessment tools, - Brochures and education materials for PCPs and patients, - Job descriptions and scope of practices for depression care team members, - Standards for assessing diagnoses, etc., and - Consult forms/procedures. |
| **Site XX identification of clinical resources** | |
| Hire or detail a nurse II (or higher) to serve as the DCM. | To be named:   - Choose between 1.0 FTE nurse II or III, - Make DCM available for 30 hours of video- and audio-based training by project YY, and - Specify DCMs’ clinical responsibilities, e.g. care management, assist with group education sessions, etc. |
| Designate clinical supervisor for DCM. | To be named:   - Meets weekly with DCM for 1 hour to review cases. |
| Identify local MH treatment resources for: cognitive behavioral therapy (CBT), grief, post-traumatic stress disorder (PTSD), substance abuse, etc. | - Assess system resources, decide where to direct particular referrals, etc. |
| Designate doctor of pharmacy (PharmD) or registered nurse practitioner (RNP) for medication management. | To be named |
| **Site XX Educational Leadership Decisions** | |
| Develop plan to train depression care team. | - Identify education leaders to arrange trainings on diagnosing depression, medication management, and dealing with unwilling patients; - Attend regional/national conferences; and - Organize supervised self-study, role playing. |
| Determine who will provide academic detailing and feedback on depression guidelines and patient outcomes. | To be named |
| **Site XX informatics needs** | |
| Tailor to system | - Adopt or adapt protocols to fit local system, and - Assist DCMs with informatics problems. |
| **Site XX performance improvement priorities** | |
| Specify involvement of quality management/improvement. | - Track DCM workload - Collect data on process outcomes, patient outcomes, provider outcomes, and performance measures. |
| Collaborate on how project YY sites will prepare for performance standards. | - Run reports to determine current performance and identify areas for improvement. |

_________________________________________________________________

Chief of Primary Care Approval/Disapproval

_________________________________________________________________

Chief of Psychiatry Approval/Disapproval

**Site XX memorandum of agreement**

1. **Contracting parties.** This memorandum of agreement (MoA) describes the work that will be provided to site XX by project YY. Site XX will be represented by YY’s depression initiative steering committee. The agreement covers materials and technical assistance required to successfully implement an effective, guideline-concordant program for the collaborative care management of persons suffering from depression.
2. **Timeline and amount.** The fee for this work is $_. This MoA covers only the time period from xx/xxxx through xx/xxxx. Payment for project YY’s services is to be made to the project YY’s institutional financial administrator.
3. **Background and parameters.** This agreement recognizes that:

A. Site XX intends to improve depression care throughout the hospital, clinic, network, and region (select as appropriate), as evidenced by its willingness to participate. Improved depression care will be based on depression guidelines and collaborative care as recognized by strategic plan or primary care directive that is appropriate to site XX’s institutional directives.

B. The depression quality improvement plan is a Knowledge Management Product for depression. (Information specific to project YY and project YY’s institutional directives should be included here.)

C. Under terms of this agreement, site XX will access the expertise of project YY to accomplish its depression improvement goals.

1. **Agreement deliverables and responsibilities**

A. Site XX will provide overall depression care improvement leadership and decision-making to its participating facilities through the depression initiative steering committee. The steering committee is responsible for:

1. Implementing the actual policies, personnel actions, and agreements -- all of which were originally developed with the participation of site XX staff through project YY.

2. Carrying out any relevant negotiations with unions, medical centers, and any staff participating in depression care improvement.

3. Providing project YY staff with de-identified, aggregated quality improvement data from sites, clinicians or individuals.

4. Disseminating project YY prepared quality improvement reports to appropriate entities.

B. Project YY will provide tools, training, and consultation required to implement collaborative care for depression. A project time allowance equivalent to approximately $- per year will be made available to site XX under this agreement.

1. Specific tools include:

- Implementation steps for sites, including engagement of primary care, mental health specialty, nursing, quality improvement, and informatics personnel, and the activities for which each must be responsible;
- Site recruitment and introductory tools and slide presentations;
- Protocol for assessing suicide risk and ensuring safety of high-risk patients;
- Depression care manager position description, and description of types of individuals who have been successfully employed as depression care managers, including the differences their backgrounds may make in program design;
- Depression care manager proficiencies and personnel evaluation forms;
- Continuously updated (at least quarterly) website that includes downloadable patient and clinician education material;
- Quick reference card templates that can be tailored and reproduced at sites;
- Care management tools, including a computerized depression registry;
- Extensively pilot-tested depression consultation and visit informatics codes, instructions for designing electronic consultations;
- Extensively pilot-tested depression assessment informatics tools and templates and
- Access to web, e-mail, or paper clinician surveys through appropriate channels to assess site readiness.

2. Training for depression care managers hired under project YY includes:

- Basic training and certificate of completion: Three days of training covering clinical informatics, assessment, education, and pro-active follow-up skills required for depression care management, completed with a final examination; and
- Ongoing training and problem identification through weekly depression care management calls.

3. Technical assistance and consultation:

- Access to depression subgroup expert consultation;
- Participation by up to three project YY staff in steering committee phone calls, no more than monthly, at the steering committee’s request;
- Access to informatics specialists who will work with sites to implement depression-specific applications, at no more than _hours per month; and
- Quarterly reports on each site’s depression care management results using de-identified, aggregate data submitted by depression care managers via the steering committee.

_________________________________________________________________

Contracting Official, Approval/Disapproval

Site XX Circle One

_________________________________________________________________

Director, Approval/Disapproval

Project YY Circle One
