# Supplementary material for: Human subjects protection issues in QUERI implementation research: QUERI Series
Source: Implement Sci. 2008 Feb 15;3:10. doi: 10.1186/1748-5908-3-10 (PMC2276514; doi:10.1186/1748-5908-3-10)
Supplement: Additional file 3 — IRB contact questionnaire. A process aid designed to simplify the communication and interaction with the IRB by centralizing some basic information on the IRB and its process and procedures. [file 1748-5908-3-10-S3.doc]

**IRB contact questionnaire**

Purpose: By using the questionnaire, the research team can confirm the forms and information that are necessary at each site for the research being conducted, learn about specific internal review board (IRB) processes, and develop a better understanding of the relationship between the IRB and the institution’s administrative mechanisms (R&D).

Relevance: The information gathered through the questionnaire can minimize communication difficulties between researchers and the IRB, and facilitate the IRB approval process.

*These process aids have been developed and refined over the course of our projects, and we anticipate they will continue to evolve over time.  Please feel free to use or adapt them to your projects as necessary.*

**IRB contact checklist**

Before engaging in conversation with an IRB, it is important to check if the IRB has a website and determine what questions can be answered, even partially, from information presented on the site. Time spent with the IRB contact can then be focused on acquiring additional information or clarification, of information already obtained.

1. Where is the best place to retrieve all necessary IRB forms – initial submission, modification, renewal, etc.?
2. When forms are updated, and how is that information disseminated?
3. What is the IRB meeting schedule?
4. Are exempt submissions reviewed by the committee at scheduled meetings or by the chair, independent of the meeting schedule?
5. What is the timeline for a submission?
6. Can research staff contact the IRB on issues relating to project YY on behalf of the site PI?
7. Can research staff be included on all correspondence to site PI?
8. How does the IRB define adverse events and serious adverse events? What are the reporting timelines and requirements for each?
