# Supplementary material for: Human subjects protection issues in QUERI implementation research: QUERI Series
Source: Implement Sci. 2008 Feb 15;3:10. doi: 10.1186/1748-5908-3-10 (PMC2276514; doi:10.1186/1748-5908-3-10)

IRB relational database

Purpose: The IRB relational database tracks all relevant paperwork and monitors deadlines, durations between submission and approval, time lapsed since submission, Site PI trainings, and almost any aspect of the IRB process. Information for all sites or specific to project, site, or region can be extracted and presented as reports. The database is especially useful to track information at sites where the component submission approach is utilized.

Relevance: Using a database for information storage can increase efficiency and improve overall levels of organization, helping avoid delays as a result of missing time-sensitive deadlines. Having this information instantly accessible can be useful for an audit.

Note: We have included broad information to start a database using Microsoft ACCESS, including suggested tables, relationships, field names and data types, and a sample form with tabs for specific aspects of the IRB process. All of these items can be modified to best fit a particular research project and don't represent the only way to organize a database or data entry form. The Leszynski/Reddick naming convention is used throughout. More information on the naming conventions can be found at: http://msdn.microsoft.com/archive/default.asp?url=/archive/en-us/dnaraccess/html/msdn_20naming.asp.

The next eight pages including the following information respectively: list of suggested tables, basic relationships between the tables, three pages listing the field names, data types and descriptions of tblMAIN, the field names, data types and descriptions of tblSubmissionInfo, and a tabbed data entry form. Tabs were created to house relevant information on an IRB, Site PI, data security, and AE/SAE/Complaint/Withdrawal information and have been circled on the form. The tab approach breaks down the information from tblMAIN and keeps the form from getting too cluttered. The Submission Info tab is a sub-report that allows users to enter multiple submission information for one site. Look-up tables were created for information that is entered repeatedly in order to ensure consistency in data entry. Using the "zoom" feature in Adobe Acrobat Reader will allow users to read pages more clearly.

These process aids have been developed and refined over the course of our projects, and we anticipate they will continue to evolve over time. Please feel free to use or adapt them to your projects as necessary.

Suggested tables

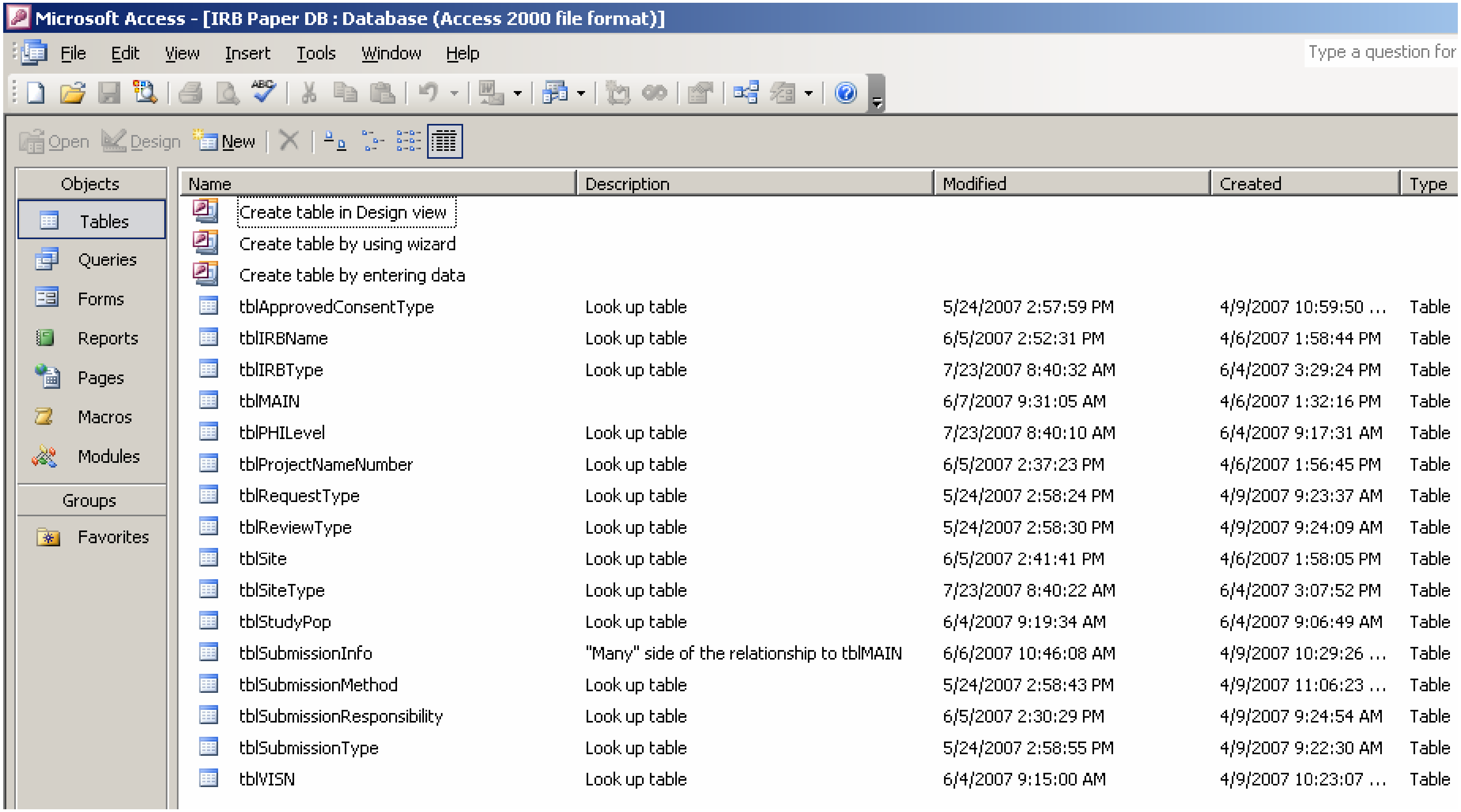

Name	Description	Modified	Created	Type
Create table in Design view				
Create table by using wizard				
Create table by entering data				
tblApprovedConsentType	Look up table	5/24/2007 2:57:59 PM	4/9/2007 10:59:50 ...	Table
tblIRBName	Look up table	6/5/2007 2:52:31 PM	4/6/2007 1:58:44 PM	Table
tblIRBType	Look up table	7/23/2007 8:40:32 AM	6/4/2007 3:29:24 PM	Table
tblMAIN		6/7/2007 9:31:05 AM	4/6/2007 1:32:16 PM	Table
tblPHILevel	Look up table	7/23/2007 8:40:10 AM	6/4/2007 9:17:31 AM	Table
tblProjectNameNumber	Look up table	6/5/2007 2:37:23 PM	4/6/2007 1:56:45 PM	Table
tblRequestType	Look up table	5/24/2007 2:58:24 PM	4/9/2007 9:23:37 AM	Table
tblReviewType	Look up table	5/24/2007 2:58:30 PM	4/9/2007 9:24:09 AM	Table
tblSite	Look up table	6/5/2007 2:41:41 PM	4/6/2007 1:58:05 PM	Table
tblSiteType	Look up table	7/23/2007 8:40:22 AM	6/4/2007 3:07:52 PM	Table
tblStudyPop	Look up table	6/4/2007 9:19:34 AM	6/4/2007 9:06:49 AM	Table
tblSubmissionInfo	"Many" side of the relationship to tblMAIN	6/6/2007 10:46:08 AM	4/9/2007 10:29:26 ...	Table
tblSubmissionMethod	Look up table	5/24/2007 2:58:43 PM	4/9/2007 11:06:23 ...	Table
tblSubmissionResponsibility	Look up table	6/5/2007 2:30:29 PM	4/9/2007 9:24:54 AM	Table
tblSubmissionType	Look up table	5/24/2007 2:58:55 PM	4/9/2007 9:22:30 AM	Table
tblVISN	Look up table	6/4/2007 9:15:00 AM	4/9/2007 10:23:07 ...	Table

Suggested relationships

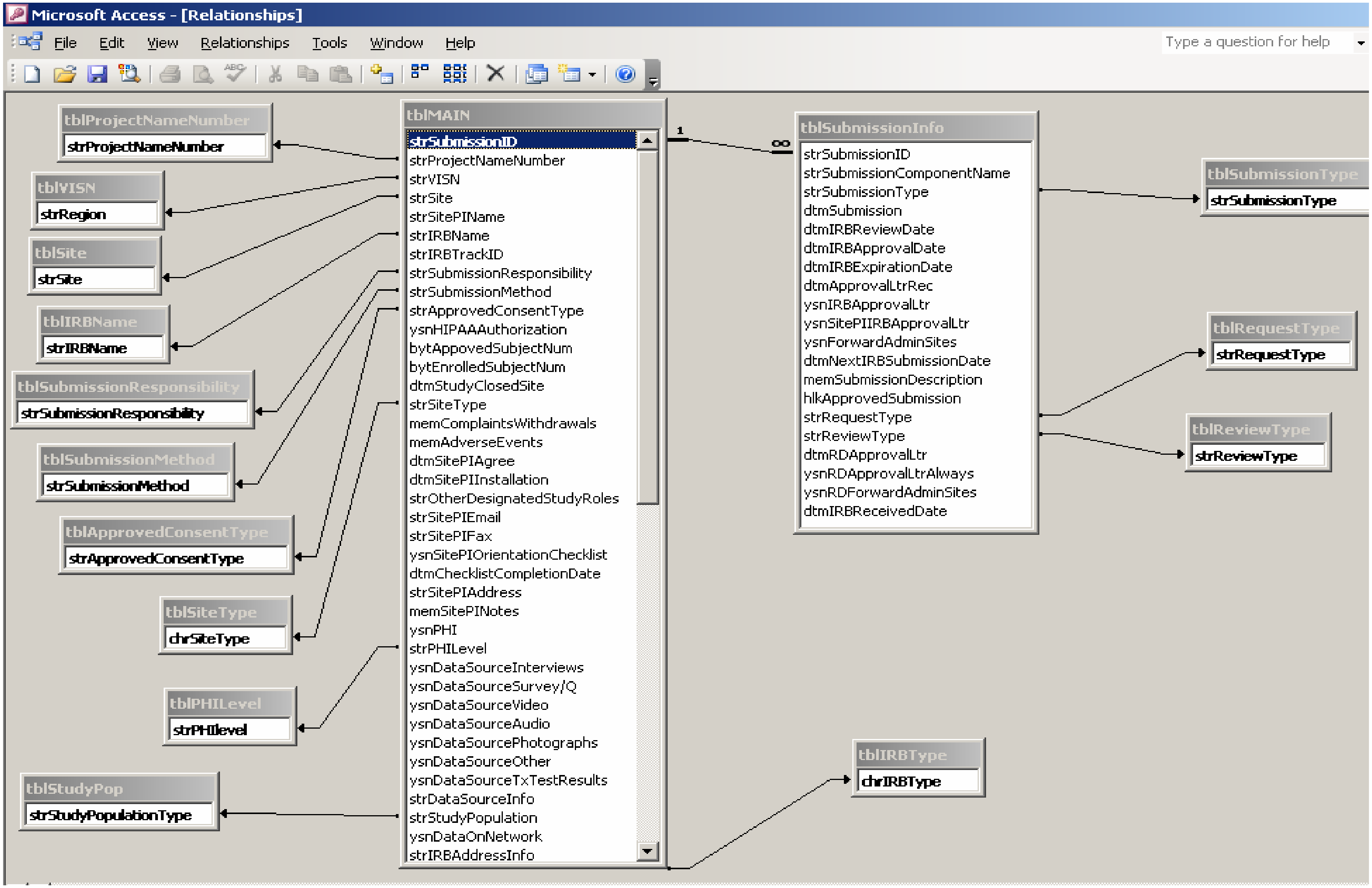

Suggested field names, data types, and descriptions of tblMAIN (1 of 3)

Microsoft Access - [tblMAIN : Table]

Field Name	Data Type	Description
strSubmissionID	Text	Created by: First letter of project, Site letter, # in consecutive order, starting with 1
strProjectNameNumber	Text	look-up field / Project name (or abbreviation) and funding #
strVISN	Text	look-up field / Geographical region
strSite	Text	look-up field / Site name
strSitePIName	Text	Complete first and last name, including suffixes
strIRBName	Text	look-up field / Official IRB name
strIRBTrackID	Text	Number/Letter string assigned to a submission by the IRB
strSubmissionResponsibility	Text	look-up field / Admin Site A, Admin Site B, etc.
strSubmissionMethod	Text	look-up field / paper, online
strApprovedConsentType	Text	look-up field / oral consent, written consent, waiver of informed consent, waiver of informed written consent
ysnHIPAAAuthorization	Yes/No	Check if there exsits HIPAA authorization from subject OR waiver of HIPAA authorization for the study has been granted by IRB
bytAppovedSubjectNum	Number	IRB approved # of subjects allowed to participate in the study AT THIS SITE
bytEnrolledSubjectNum	Number	# of consented, enrolled subjects AT THIS SITE
dtmStudyClosedSite	Date/Time	Date study was closed AT THIS SITE (project may still remain open at other sites)
strSiteType	Text	look-up field
memComplaintsWithdrawals	Memo	Detail any information on complaints or withdrawals involving this site
memAdverseEvents	Memo	Detail any information on Adverse Events or Serious Adverse Events involving this site
dtmSitePIAgree	Date/Time	Date Site PI agreed to Site PI responsibilities. Not always formal. For example, may occur via email.
dtmSitePIInstallation	Date/Time	Date Site PI has completed all required trainings and other responsibilities and is able to officially be Site PI
strOtherDesignatedStudyRol	Text	Any additional roles the Site PI may be taking on in conjunction with being Site PI?
strSitePIEmail	Text	Email address for Site PI
strSitePIFax	Text	Fax number for Site PI
ysnSitePIOrientationChecklist	Yes/No	Has the Site PI Orientation Checklist been completed?
dtmChecklistCompletionDate	Date/Time	Date Site PI Orientation Checklist completed.
strSitePIAddress	Memo	Insert all address info. Using text box approach for easy cut & paste.
memSitePINotes	Memo	Notes relating to Site PI

Suggested field names, data types, and descriptions of tblMAIN (2 of 3)

Microsoft Access - [tblMAIN : Table]

Field Name	Data Type	Description
ysnPHI	Yes/No	Check if any level of PHI be utilized, accessed, collected, or generated as a part of the study
strPHILevel	Text	Select the most stringent level of PHI being used in the study. de-identified; limited; fully identifiable
ysnDataSourceInterviews	Yes/No	Provide more detail on this selection under "Data Source Info" textbox in form
ysnDataSourceSurvey/Q	Yes/No	Provide more detail on this selection under "Data Source Info" textbox in form
ysnDataSourceVideo	Yes/No	Provide more detail on this selection under "Data Source Info" textbox in form
ysnDataSourceAudio	Yes/No	Provide more detail on this selection under "Data Source Info" textbox in form
ysnDataSourcePhotographs	Yes/No	Provide more detail on this selection under "Data Source Info" textbox in form
ysnDataSourceOther	Yes/No	Provide more detail on this selection under "Data Source Info" textbox in form
ysnDataSourceTxTestResults	Yes/No	Provide more detail on this selection under "Data Source Info" textbox in form
strDataSourceInfo	Memo	Brief description of data source info and PHI levels. (interviews, surveys, medical records, audio, tissue samples, test results)
strStudyPopulation	Text	Select one: VA; Non-VA; Both VA and non-VA
ysnDataOnNetwork	Yes/No	Check if all project data is stored on HSRD network, behind firewall
strIRBAddressInfo	Memo	Insert all address info. Using text box approach for easy cut & paste.
bytIRBPhone1	Text	Phone number of IRB
bytIRBPhone1Ext	Text	Extension of IRB Phone 1 (if applicable)
bytIRBPhone2	Text	Alternate phone number of IRB
bytIRBPhone2Ext	Text	Extension of IRB Phone 2 (if applicable)
bytIRBFax	Text	Fax number for IRB
strIRBGeneralEmail	Text	General email address of IRB
hlkIRBwebsite	Hyperlink	Official IRB website
strIRBMtgSchedule	Text	General schedule of IRB meeting. I.e.: Every Wednesday? 1st & 3rd Fridays of the month?
strIRBSubmissionDeadline	Text	General submission deadline as determined by IRB. Generally several weeks prior to expiration. I.e.: 2 weeks, 6 weeks, 8 weeks, etc.
strIRBType	Text	look-up field - what kind of IRB? VA-in house; University affiliated; Other-VA
strrIRBContactFirst	Text	First name of IRB Contact - may or may not be Chair or Administrator
strIRBContactLast	Text	Last name of IRB Contact
strIRBContactTitle	Text	Title of IRB Contact

Suggested field names, data types, and descriptions of tblMAIN (3 of 3)

Microsoft Access - [tblMAIN : Table]

File Edit View Insert Tools Window Help

Field Name	Data Type	Description
strIRBContactPhone	Text	Phone number of IRB Contact
strIRBContactPhoneExt	Text	Extension of IRB Contact Phone (if applicable)
strIRBContactEmail	Text	Personal email address of IRB Contact
memNotes	Memo	Additional information
ysnIRBContactChecklist	Yes/No	Has the IRB Contact Checklist been completed?
ysnStaffforPI	Yes/No	Can designated research staff contact the IRB on behalf of the Site PI?
chrIRBApprovalIncludes	Text	What does IRB approval? Just VAMC? VAMC and CBOCs? Which CBOCs?
intComplaints	Number	# of complaints at this site
intWithdrawals	Number	# of subject withdrawals at this site
intAEs	Number	# of adverse events at this site
strRDContactFirst	Text	First name of R&D Contact - may or may not be Chair/Administrator
strRDContactLast	Text	Last name of R&D Contact
strRDContactPhone	Text	Phone number of R&D Contact
strRDContactPhoneExt	Text	Extension of R&D Contact Phone (if applicable)
strRDContactEmail	Text	Personal email address of R&D Contact
strRDAddress	Text	Insert all address info. Using text box approach for easy cut & paste.
bytRDFax	Text	Fax number for R&D Committee
strRDMeetingSchedule	Text	General schedule of R&D meeting. I.e.: Every Wednesday? 1st & 3rd Fridays of the month?
strRDSubmissionDeadline	Text	General submission deadline as determined by R&D Committee.
strRDChair	Text	Who is the R&D Committee Chair?
memRDNotes	Memo	Any additional information on R&D Committee & process not already captured
strRDApprovalLtrPolicy	Text	Notes on how and when this R&D generates approval letters

Suggested field names, data types, and descriptions of tblSubmissionInfo

Microsoft Access - [tblSubmissionInfo : Table]

File Edit View Insert Tools Window Help   Type a question for hel

Field Name	Data Type	Description
strSubmissionID	Text	This foreign key connects to the chrID primary key in table MAIN
strSubmissionComponentNam	Text	Name of component submission - short & brief!
strSubmissionType	Text	look-up field / component; initial; modification-amendment; renewal
dtmSubmission	Date/Time	Date submitted to IRB
dtmIRBReviewDate	Date/Time	Date reviewed by IRB - usually indicated by IRB meeting schedule
dtmIRBApprovalDate	Date/Time	Date approved by IRB - officially indicated on IRB submission with signature from IRB chair
dtmIRBExpirationDate	Date/Time	Date IRB approval will expire - officially indicated on IRB submission
dtmApprovalLtrRec	Date/Time	Date study staff received IRB approved submission
ysnIRBApprovalLtr	Yes/No	Check if IRB approval letter has been received by study staff
ysnSitePIIRBApprovalLtr	Yes/No	Check if Site PI has received a copy of the IRB Approval Letter
ysnForwardAdminSites	Yes/No	Check if IRB information has been forwarded to other administrative sites
dtmNextIRBSubmissionDate	Date/Time	Date of next IRB required submission
memSubmissionDescription	Memo	For any additional submission information
hlkApprovedSubmission	Hyperlink	Scan signed, approved submission into a PDF & hyperlink
strRequestType	Text	look-up field / exempt; expedited; full committee
strReviewType	Text	look-up field / exempt; expedited; full committee
dtmRDApprovalLtr	Date/Time	Date of R&D Approval
ysnRDApprovalLtrAlways	Yes/No	Is an R&D approval letter (and date) generated for every IRB submission (initial, modification, renewal, etc.)? If yes, enter date as well.
ysnRDForwardAdminSites	Yes/No	Check if R&D approval letter has been forwarded to other administrative sites (applies only if R&D letter has been generated)
dtmIRBReceivedDate	Date/Time	Date submission was received by IRB (usually stamped on application, or noted in the approval letter)

Suggested tabbed data entry form

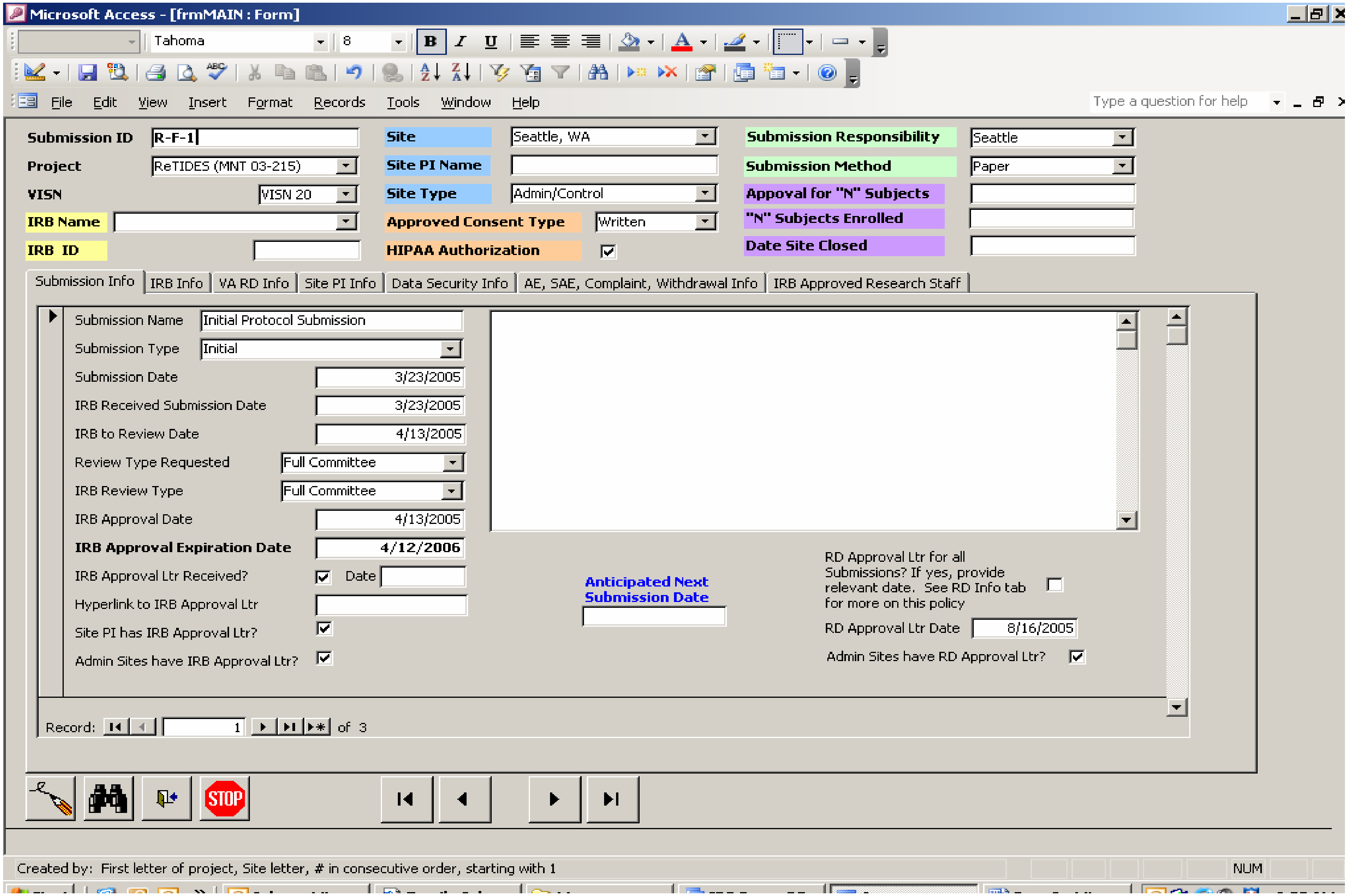

Supplement: Additional file 4 — IRB Relational Database. A process aid which is an example of how a relational database can be used to assist with IRB tracking across sites. [file 1748-5908-3-10-S4.pdf]
