# Supplementary material for: Human subjects protection issues in QUERI implementation research: QUERI Series
Source: Implement Sci. 2008 Feb 15;3:10. doi: 10.1186/1748-5908-3-10 (PMC2276514; doi:10.1186/1748-5908-3-10)
Supplement: Additional file 5 — Site PI orientation checklist. A process aid to collect contact information and preference for the site PI as well as a tool for discussing IRB responsibilities and division of labor. [file 1748-5908-3-10-S5.doc]

**Site PI orientation checklist**

Purpose: The checklist can be used to gather necessary information from a site PI and to discuss procedures regarding internal review board (IRB) submissions, file retention, and adverse events. This information also can be entered into the IRB relational database for future queries and reports.

Relevance: The checklist can orient the site PI to responsibilities, ensure research staff provide as much administrative support as necessary, ensure smooth operation of the research protocol, and enhance the site PI’s overall experience and success.

*These process aids have been developed and refined over the course of our projects, and we anticipate they will continue to evolve over time.  Please feel free to use or adapt them to your projects as necessary.*

**Site PI orientation checklist**

**General information**

1. Site name and type
2. Site PI complete first and last name
3. Degree(s)
4. Title(s)
5. Preferred salutation and name (note any nicknames)
6. Preferred work mailing address
7. Is FedEx mailing address, same as above?
8. Email address(es) (for multiple addresses, determine preference)
9. Phone number(s) (for multiple numbers, determine preference)
10. Time zone
11. Pager number
12. Fax number
13. Assistant? (If yes, collect assistant information)

- Assistant name
- Assistant e-mail (include on all e-mails to site PI?)

1. Should mail correspondence be sent to Site PI or Site PI Assistant?
2. Days or times when Site PI not available due to clinic, teaching, surgery, etc.?
3. Site PI contact preference
4. Provide site PI with contact information for project YY research staff, including phone and fax numbers, e-mail, and address

**Study information**

1. What is the site PI familiarity with project YY? Is the site PI aware of other staff participating in project YY at his/her site? Would the site PI like additional background information on project YY?
2. Discuss site PI role in project YY, specifically what research activities will be carried out at his/her site.
3. Present current opportunities for site PIs in project YY. Are there topics the site PI would like to be involved in (i.e., manuscripts, presentations, work groups, etc.)?

**IRB information**

1. Confirm IRB of record. Is site PI familiar with location?
2. Has the site PI submitted other proposals to this IRB before? If yes, are there recommended contact personnel?
3. Will the site PI correspond with the IRB directly or, if possible, have project YY research staff correspond with the IRB and update the site PI as necessary?
4. Discuss IRB submission process. How would site PI prefer to receive completed applications for submission? Via e-mail? Via FedEx?
5. Discuss project YY adverse events procedure.
6. Discuss IRB records retention requirements and procedure should an audit take place.

**Documentation information**

1. How will the site PI submit IRB documents, i.e. hand delivery, interoffice mail, etc.? Should research staff include forwarding envelopes (and postage) to facilitate submission?
2. Will site PI make copies of the IRB submission for his/her files, or should research staff send a copy?
3. If site PI prefers to have copies mailed, would he/she like the information sent in labeled file folder?

**Ethics certification requirements**

1. What ethics trainings are required for researchers at the site PI’s site?
2. Is the site PI current on the required trainings?
3. When will the trainings expire?
4. Would the site PI like a reminder from research staff prior to the training expirations?
