# Supplementary material for: Human subjects protection issues in QUERI implementation research: QUERI Series
Source: Implement Sci. 2008 Feb 15;3:10. doi: 10.1186/1748-5908-3-10 (PMC2276514; doi:10.1186/1748-5908-3-10)
Supplement: Additional file 6 — IRB specialist position description. a process aid job description including how having a designated person in this role can be helpful. [file 1748-5908-3-10-S6.doc]

### IRB specialist position description

### Purpose: The position description outlines the duties and responsibilities of the internal review board (IRB) specialist.

### Relevance: The IRB specialist can aid in navigating the challenges of implementation research. Parts of the position description can be used in grant writing to justify budget expenditures for an IRB Specialist.

### Note: This position is officially titled “Pre-Awards Research Administrator/IRB Coordinator,” but was shortened to “IRB Specialist” in the manuscript.

### *These process aids have been developed and refined over the course of our projects, and we anticipate they will continue to evolve over time.  Please feel free to use or adapt them to your projects as necessary.*

### IRB specialist

## Major duties

This individual’s primary role will be to coordinate pre-award activities, which include proposal submissions, preparation of budgets, and ensuring compliance with pre-award regulations and guidelines. This individual will oversee and coordinate the implementation and management of the Institutional Review Board (IRB) related efforts for Health Services Center studies.

The IRB specialist position is an important role due to the rapid growth of health services research among investigators, the increasing complexity of the IRB application process, and HIPAA (Health Insurance Portability and Accountability Act) requirements. The specialist is expected to work with institutional research and development (R&D), health services center leadership, regional institute for biomedical and clinical research, and regional university IRB for the efficient and effective management of proposal and IRB submissions for all studies. The specialist will oversee program staff in the preparation of IRB submissions, grant proposals, and presentations of workshops and trainings.

**Resource:** The IRB specialist will assist center investigators in the preparation of grant proposals, applications to the IRB, and informed consent forms. This includes guidance regarding HIPAA requirements (data use agreements, business associate agreements), Quality assurance (QA) vs. research determination, consent procedures, forms, and the categories of research that are exempt from committee review or that qualify for expedited review. The specialist will maintain two electronic guides specific to IRB and HIPAA, and grant proposal preparation and submission issues that can be used for guidance in preparing applications and proposals. The specialist also will maintain an electronic guide to specific IRB and HIPAA issues (i.e., when exemption is appropriate, and access to datasets with and without patient identifiers), with concrete examples of IRB applications and how they have addressed those issues. This individual also will conduct educational sessions for Center investigators, project staff, and other research support personnel to increase knowledge and awareness of human subject and HIPAA issues.

**Systems**: The IRB specialist will maintain systems and procedures to assist Center investigators and staff with IRB and HIPAA compliance including electronic guides and a data base of data use agreement and business associate agreements, as well as QA vs. Research determinations.

**Liaison:** The specialist will serve as the center liaison for health services-related IRB and HIPAA issues and grant proposal submissions to groups such as the institutional R&D, regional institute for biomedical and clinical research, human subjects division at the regional university, the regional multi-site IRB, and the regional data warehouse advisory committee. This individual will proactively present updates and briefings to center leadership on anticipated IRB issues.

**Proposal Preparation:** The specialist will oversee and supervise program staff in the preparation and submission of center grant proposals. For example, the specialist may help develop the budget, Gantt charts, and consent procedures. This individual will provide access to and assist in interpreting federal regulations in proposal preparations, issue notices related to updated proposal guidelines, and conduct proposal submission workshops.

## FACTOR 1. Knowledge required by the position

This individual possesses professional knowledge as evidenced by a master’s degree with two years applicable work experience OR equivalent education/experience in a related health services field.

The IRB specialist will develop in depth knowledge of IRB, HIPAA, and other regulations pertinent to the participation of human subjects in health services research. The IRB specialist also should be cognizant of guidelines and regulations applicable to proposal submissions for federally-funded, non-federally funded, and privately-funded research studies.

## FACTOR 2. Supervisory controls

This individual reports to the health services center associate director. The health services center and R&D administrative officers provide administrative guidance and assistance. The various principal investigators and project directors who determine research objectives and priorities provide general supervision with regard to activities directly related to their individual projects. The incumbent acts independently in carrying out tasks relevant to IRB and HIPAA compliance.

## FACTOR 3. Guidelines

Guidelines include the objectives as specified in the project proposal, HIPAA regulations, institutional and regional university regulations concerning the use of human subjects, and institutional central office guidelines for proposal submission and human subjects protection. This individual is called upon to apply his/her training and experience in developing strategies for successful goal attainment. Independent judgment and creativity are required to interpret and apply general guidelines on accepted scientific practice. Administrative guidelines are available in the form of site memoranda, health services center and institutional R&D memoranda, regional university IRB regulations, HIPAA regulations, and institutional central office directives and regulations.

## FACTOR 4. Complexity

This individual provides technical guidance and performs complex duties to facilitate IRB and grant proposal submission process and submissions. This involves use of judgment in independent interpretation and application of relevant federal and state laws, regulations, institutional policies, and guidelines.

## FACTOR 5. Scope and effect

This individual is expected to work jointly with the regional university IRB and center leadership and assist in the efficient and effective management of IRB submission, review, approval, renewal, and closeout procedures affecting health services center letters of intent, proposals, and studies.

## FACTOR 6. Personal contacts

Local contacts are with the entire health services center staff including director and associate director, administrative staff, principal investigators, project directors, collaborating investigators, clinicians and administrators including privacy officers at various sites. Other contacts include IRBs at other affiliated institutions, investigators at other institutions, the regional data warehouse advisory committee, and the regional university grant and contracts department, and human subjects division.

## FACTOR 7. Purpose of the contacts

Contacts with the local health services center staff are to facilitate compliance with institutional policies, HIPAA regulations and to facilitate the development and submission of IRB applications to the regional university IRB. Contact with the regional institute for biomedical and clinical research is to assure the timely review and feedback from the human subjects division to assure paperwork is complete and ready for proposal funding.

## FACTOR 8. Physical demands

The work is sedentary in nature with occasional travel between institutional and regional university locations. Incumbent possibly will be required to attend training sessions or conference in the local area or across the United States. Physical mobility and moderate strength are required as some short‑term tasks may require lifting of objects weighing forty pounds or less.

## FACTOR 9. Work environment

Work is performed in an office or clinic setting with significant amounts of time on the telephone and using a computer workstation.

## FACTOR 10. Safety

It is the incumbent's responsibility to be safety conscious, and to be alert and to recognize hazardous conditions and do what is possible to eliminate or assist in eliminating these conditions, or, if that cannot be done, to work safely with them; to avoid committing unsafe acts; and to be watchful of the safety of other employees for mutual protection. The incumbent is to report unsafe working conditions immediately to the supervisor and is encouraged to apply his/her training and experience in planning better methods and arrangements.

## FACTOR 11. Security

The incumbent protects printed and electronic files containing sensitive data in accordance with the provisions of the Privacy Act of 1974, and other applicable laws, federal regulations, institutional statutes and policy. The incumbent also protects the data from unauthorized release, or from loss, alteration, or unauthorized deletion, and follows applicable regulations and instructions regarding access to computerized files, release of access codes, etc., as set out in the computer access agreement that the employee signs.
