# Supplementary material for: Human subjects protection issues in QUERI implementation research: QUERI Series
Source: Implement Sci. 2008 Feb 15;3:10. doi: 10.1186/1748-5908-3-10 (PMC2276514; doi:10.1186/1748-5908-3-10)
Supplement: Additional file 7 — IRB Call Minutes Template. A template/process aid which can be used to record the discussion and particularly the decisions made during IRB calls. [file 1748-5908-3-10-S7.doc]

# IRB Call Minutes Template

#

# Purpose: Capturing minutes of IRB-specific conference calls has several benefits including

# sharing information with those not in attendance, noting decisions made, and ensuring a record

# of division of responsibilities.

# Relevance: Communication among the research staff is important for coordination, consistency, and resolution of IRB issues.

# Note: Example agenda items from previous IRB calls have been included in the template and

# can be adjusted to best suit a particular project.

#

# *These process aids have been developed and refined over the course of our projects, and we anticipate they*

# *will continue to evolve over time.  Please feel free to use or adapt them to your projects as necessary.*

# IRB Call MINUTES

| Xx / xx / xxxx | Alphabetical listing of call participants. | | |
| --- | --- | --- | --- |
| Discussion | | Follow-up |  |
| 1. Submission Deadlines  - Insert text … | | - Insert follow-up plans … |  |
| 2. Upcoming Renewal Deadlines | |  |  |
| 3. Internal Audit of administrative site IRB files | |  |  |
| 4. Site PI Tracking Responsibilities | |  |  |
| 5. Manuscript Update | |  |  |
| 6. Future Grant – IRB Considerations & Questions | |  |  |
| 7. Call Schedule | |  |  |
| 8 Project YY Activities Update | |  |  |
| 9. Other | |  |  |

**NEXT CALL: Xx / xx / xxxx**

Noon PST – 1:00pm MST – 2:00PM CST – 3:00PM EST

**1-800-555-5555 / Access code 55555**
